# Correlations of Online Search Engine Trends With Coronavirus Disease (COVID-19) Incidence: Infodemiology Study

**DOI:** 10.2196/19702

**Published:** 2020-05-21

**Authors:** Thomas S Higgins, Arthur W Wu, Dhruv Sharma, Elisa A Illing, Kolin Rubel, Jonathan Y Ting, Snot Force Alliance

**Affiliations:** 1 Department of Otolaryngology-Head and Neck Surgery and Communicative Disorders University of Louisville Louisville, KY United States; 2 Rhinology, Sinus & Skull Base Kentuckiana Ear Nose Throat Louisville, KY United States; 3 Department of Otolaryngology-Head and Neck Surgery Cedars Sinai Medical Center Los Angeles, CA United States; 4 Department of Otolaryngology-Head and Neck Surgery Indiana University Indianapolis, IN United States; 5 Snot Force KY United States

**Keywords:** COVID-19, coronavirus, big data, infodemiology, infoveillance, Baidu, SARS-CoV-2, Google Trends, digital health, epidemiology, China, Italy, Spain, New York, Washington

## Abstract

**Background:**

The coronavirus disease (COVID-19) is the latest pandemic of the digital age. With the internet harvesting large amounts of data from the general population in real time, public databases such as Google Trends (GT) and the Baidu Index (BI) can be an expedient tool to assist public health efforts.

**Objective:**

The aim of this study is to apply digital epidemiology to the current COVID-19 pandemic to determine the utility of providing adjunctive epidemiologic information on outbreaks of this disease and evaluate this methodology in the case of future pandemics.

**Methods:**

An epidemiologic time series analysis of online search trends relating to the COVID-19 pandemic was performed from January 9, 2020, to April 6, 2020. BI was used to obtain online search data for China, while GT was used for worldwide data, the countries of Italy and Spain, and the US states of New York and Washington. These data were compared to real-world confirmed cases and deaths of COVID-19. Chronologic patterns were assessed in relation to disease patterns, significant events, and media reports.

**Results:**

Worldwide search terms for shortness of breath, anosmia, dysgeusia and ageusia, headache, chest pain, and sneezing had strong correlations (*r*>0.60, *P*<.001) to both new daily confirmed cases and deaths from COVID-19. GT COVID-19 (search term) and GT coronavirus (virus) searches predated real-world confirmed cases by 12 days (*r*=0.85, SD 0.10 and *r*=0.76, SD 0.09, respectively, *P*<.001). Searches for symptoms of diarrhea, fever, shortness of breath, cough, nasal obstruction, and rhinorrhea all had a negative lag greater than 1 week compared to new daily cases, while searches for anosmia and dysgeusia peaked worldwide and in China with positive lags of 5 days and 6 weeks, respectively, corresponding with widespread media coverage of these symptoms in COVID-19.

**Conclusions:**

This study demonstrates the utility of digital epidemiology in providing helpful surveillance data of disease outbreaks like COVID-19. Although certain online search trends for this disease were influenced by media coverage, many search terms reflected clinical manifestations of the disease and showed strong correlations with real-world cases and deaths.

## Introduction

The coronavirus disease (COVID-19) is the most recent pandemic to occur in the digital age. The zoonotic infections influenza H5H1 in 1997 and severe acute respiratory syndrome (SARS) in 2002 led to significant interests in using advances in technology and data harvesting to assist in disease prediction, surveillance, and mitigation [1]. In 2003, Eysenbach discussed the use of population health tools and technologies, including the internet, during the 2002-2004 SARS outbreak. His work in the field has led to the concept of information epidemiology, which has been termed infodemiology [2,3]. With online search engines harvesting large amounts of data from the general population in real time and providing the information publicly, interest has risen in the potential for public health use of these data during impending outbreaks [4-10].

Google Trends (GT) and the Baidu Index (BI) are examples of Big Data surveillance tools that were developed to help researchers analyze temporal and geographical trends in online search terms or topics through the Google and Baidu search engines, respectively [11,12]. In a recent systematic review, Mavragani et al [13] identified over 100 peer-reviewed papers studying health-related phenomena using GT data, demonstrating trending in search volumes with time related to the population’s increased use of the internet search engines in seeking information regarding their health. In 2010, Zhou and Shen [14] reported that Baidu search queries and news articles were 10-40 days ahead of official epidemiology for several infectious diseases in China.

With the time stamping of these searches, we can also correlate timing of searches to major public events, media coverage, and confirmed disease spread, and possibly forecast dissemination of disease from these events. The purpose of this study was to apply this type of digital epidemiology to the current COVID-19 pandemic to determine its utility to public health surveillance efforts.

## Methods

### Region Selection

In selecting the regions, the authors chose the initial epicenter of the pandemic (China) as well as the most severely affected regions in Europe and the United States. Up to April 6, 2020, the two most affected countries in Europe were Italy and Spain with 130,759 and 128,948 confirmed cases and 15,889 and 12,418 confirmed deaths, respectively.

### Real-World Databases

Real-world data for daily confirmed cases and deaths were obtained using the World Health Organization’s (WHO) COVID-19 Dashboard for worldwide, China, Italy, and Spain, and the corresponding state department’s databases for the states of Washington and New York [15-17]. These data were normalized to a scale from 0 to 100 to allow comparisons with the search terms.

### Search Query Databases

GT [11] is a public sampling database of actual search requests performed using the Google search engine [18] that are anonymized, categorized, and aggregated. According to Google [19]: “GT normalizes search data to make comparisons between terms easier. Each data point is divided by the total searches of the geography and time range it represents to compare relative popularity. The resulting numbers are then scaled on a range of 0 to 100 based on a topic’s proportion to all searches on all topics.” Therefore, a value of 100 means the maximum search interest for the time and location selected.

The BI [12] is a public sampling database of search queries users entered into the Baidu search engine [20], the predominant search engine in China. BI is catered towards an exclusively Mandarin speaking and reading clientele, as there are no options to change language. Unlike GT, BI results are not displayed as normalized values and, instead, reflect the absolute Baidu search volume but are not equivalent to it [21]. Because of this function, results for different terms can be compared to each other for relative frequency, even across different time periods. Search terms were translated into traditional Chinese characters. BI allows for combined searches that display the results of multiple search terms added together, which can be accomplished in the search bar using “+” or by using the advanced search option. This was used when there were multiple potential words or phrases for symptoms. Unfortunately, data cannot be as conveniently extracted from BI as from GT; there is no way to download data files for search queries. However, scrolling over the search trend curve yields daily search volumes, and these search volumes were manually recorded for each search term over the studied time period.

### Disease Nomenclature and Symptom Search Term Selection

The authors selected search terms for the database query using a systematic approach. Key concepts were presented to the research group, and a preliminary list of search terms was compiled using COVID-19 nomenclature and symptomatology [22-27].

The authors used a combination of literature review, clinical experience, google searches, and news resources to compile a list of potential symptomatology associated with COVID-19. Since multiple iterations of a word may be used to search the same condition or symptom (eg, myalgia and muscle ache), GT groups a cluster of search terms as a topic or disease [28]. Therefore, topics or diseases were used over an individual search term when applicable. The list of symptom terminology considered were: fever (medical condition), shortness of breath (disease), cough (disease), anosmia (topic), fatigue (medical condition), rhinorrhea (medical condition), nasal congestion (syndrome), sneeze (topic), myalgia (topic), sore throat (topic), diarrhea (topic), anorexia (symptom), chest pain (syndrome), sputum (sputum), headache (medical condition), nausea (disorder), ageusia (topic), abdominal pain (syndrome), dizziness (medical condition), vomiting (ailment), and eye pain (topic).

Disease terminology assessed included coronavirus (virus), coronavirus (search term), COVID-19 (search term), SARS-CoV-2 (severe acute respiratory syndrome coronavirus 2; search term), and severe acute respiratory syndrome (disease). Because the timing of the nomenclature designations overlapped with the study period, we elected to study both clustered terms and individual search terms for COVID-19. The period studied was set from January 9, 2020, to April 6, 2020, to capture the last 3 months.

GT data for each symptom were obtained and compared using a Pearson correlation with the disease terms. Those terms reaching statistically significant correlations were then used in the final modeling. Two physicians fluent in Chinese determined search terms related to COVID-19 nomenclature and symptomatology for use in the BI.

Locations chosen for analysis were selected from early epicenters of the COVID-19 pandemic with reported internet search data, reported cases, and deaths available during this period. China, Wuhan (China), Italy, Spain, Washington State (United States), and New York State (United States) were selected for regional analysis. The WHO was first informed of a pneumonia-like illness outbreak in Wuhan, China on December 31, 2020. Other regions of the world then gradually started reporting their first confirmed cases, including the state of Washington (United States) on January 21, 2020, Italy and Spain on January 31, 2020, and the state of New York (United States) on March 1, 2020. Regional GT data were collected from the date of the first confirmed case.

### Significant Events

**Figure 1:**
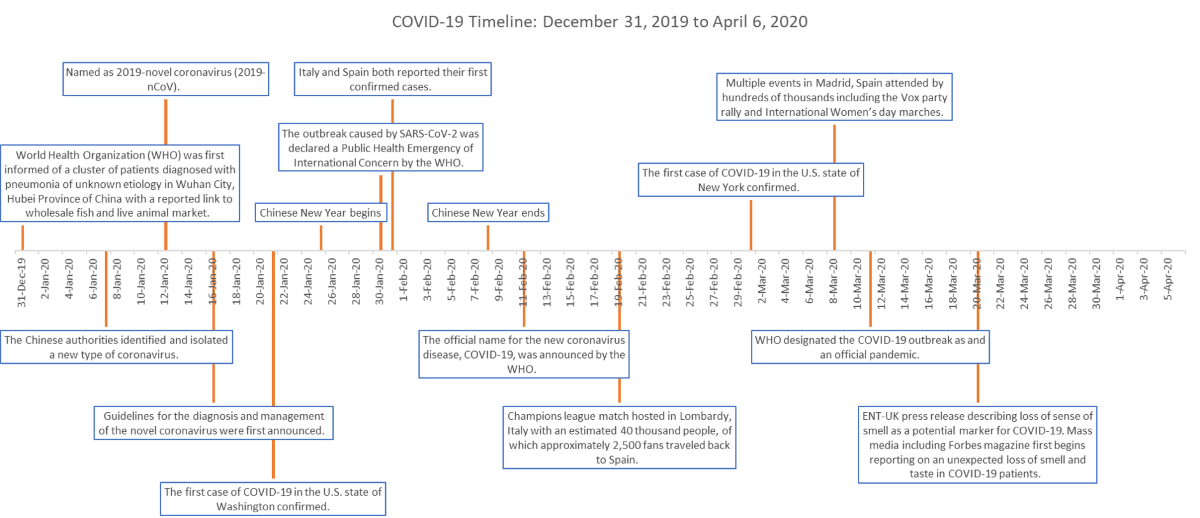
Timeline of real-world COVID-19 significant events. COVID-19: coronavirus disease; ENT-UK: British Association of Otolaryngologists; SARS-CoV-2: severe acute respiratory syndrome coronavirus 2.

The timeline of the pandemic was then outlined based upon WHO reporting of global cases around the world, as well as identification of large public events and media publications on COVID-19-related topics (Figure 1) [27,29-33]. These important dates were then compared to GT and Baidu search trends, to identify possible “super-spreader” events, media influence, and context for the trends.

### Analyses

Analyses were performed using SPSS Statistics for Windows (Version 26.0, IBM Corp). A Pearson correlation was used to compare volumes of real-world confirmed cases, real-world deaths, COVID-19 disease nomenclature searches, and symptom terms searches. Associations across time series were assessed by fitting autoregressive integrated moving average (ARIMA) models to the individual search volumes and real-world time series, based on the methods of Box and Jenkins [34]. The models were created with assessments of trend, seasonal differencing, and outliers. Autocorrelation functions and partial autocorrelation functions were assessed, and the Ljung-Box statistic was used to examine the residuals from the time series models to evaluate the lack of fit [35]. Sample cross-correlation functions (CCF) were then used to compare the time series models to assess the correlation between the explanatory and dependent time series. Lags of the time series were determined by comparing asynchronous cross-correlations and synchronous cross-correlations [36]. Significance was determined using a two-tailed *P*<.05. 

## Results

### Worldwide Real-World COVID-19 Data and GT

**Figure 2:**
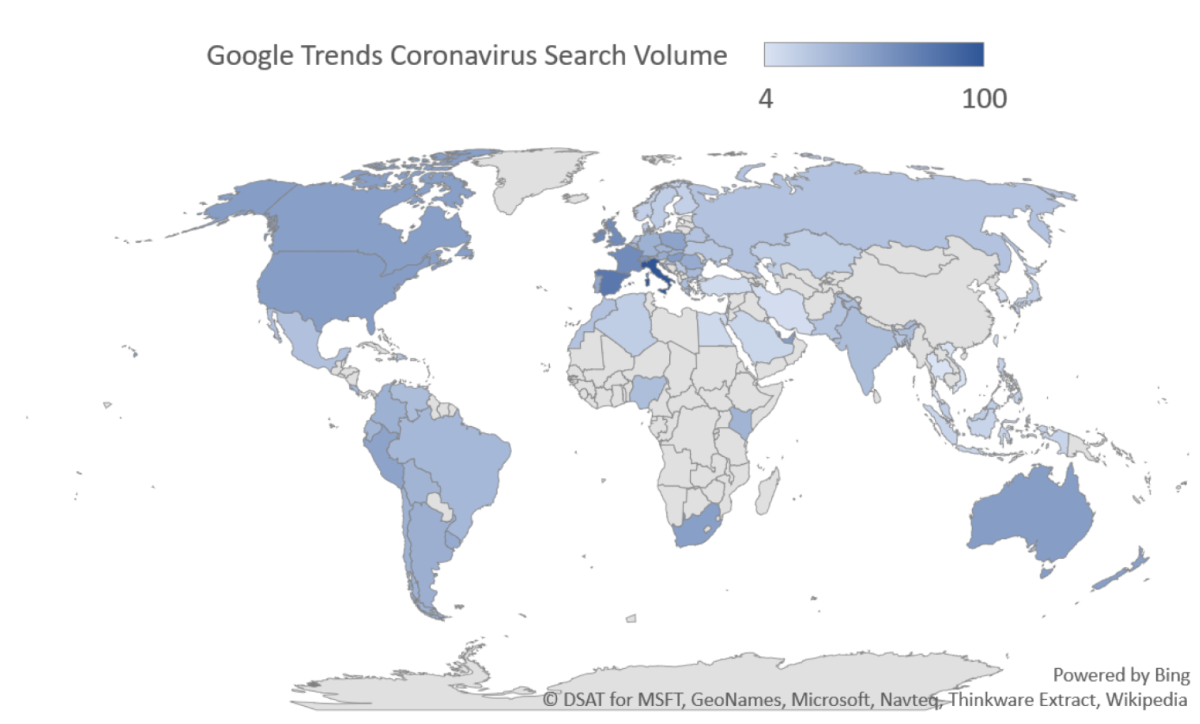
Geographic heat map of worldwide online Google searches for coronavirus (virus) between January 9, 2020, and April 6, 2020.

**Figure 3:**
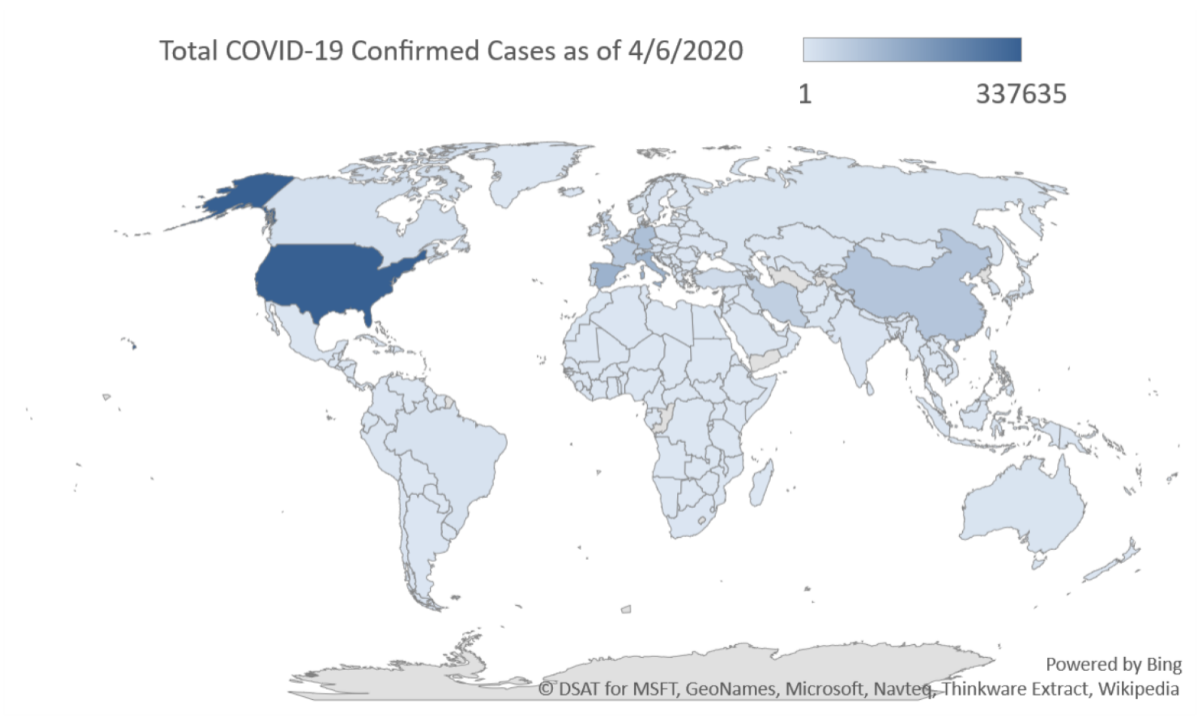
Geographic heat map of worldwide real-world confirmed cases of COVID-19 as of April 6, 2020. COVID-19: coronavirus disease.

**Figure 4:**
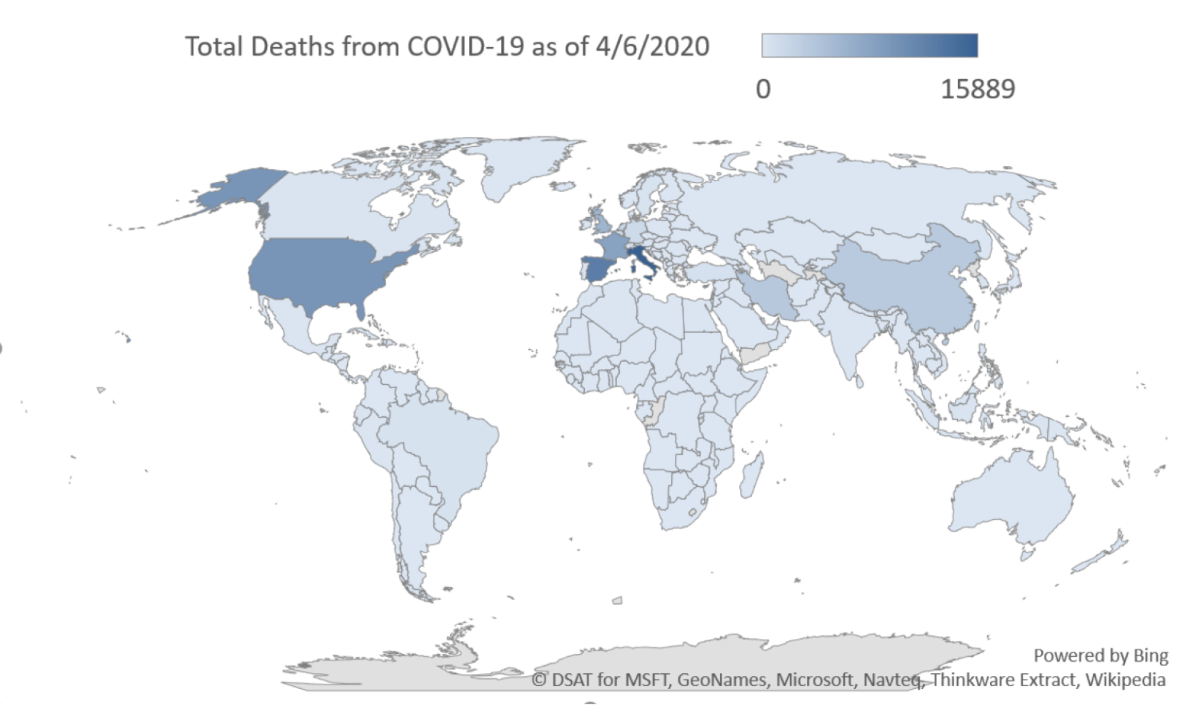
Geographic heat map of worldwide real-world deaths from COVID-19 as of April 6, 2020. COVID-19: coronavirus disease.

**Figure 5:**
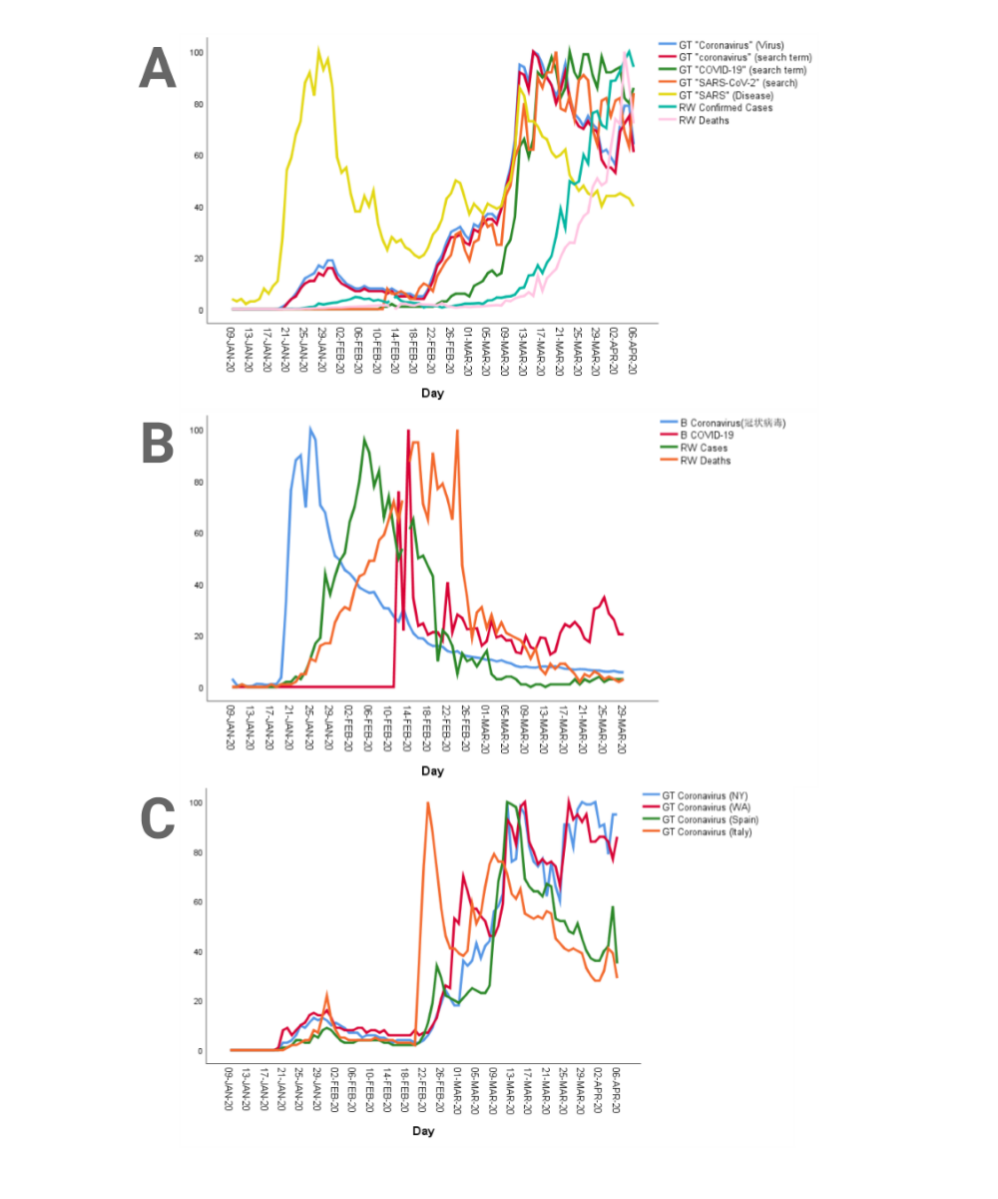
Normalized Google Trends and Baidu Index search terms by date compared to real-world new confirmed cases and deaths from COVID-19: (A) RW worldwide data and GT COVID-19 search terms, (B) China RW data and Baidu Index COVID-19 search terms, and (C) GT search for coronavirus (virus) by geographic region. B: Baidu Index; COVID-19: coronavirus disease; GT: Google Trends; RW: real-world; SARS: severe acute respiratory syndrome; SARS-CoV-2: severe acute respiratory syndrome coronavirus 2.

Figure 2 shows a geographic heat map of online Google searches for coronavirus (virus) during the study period of January 9, 2020, to April 6, 2020, which demonstrates the highest search volumes in Italy with high search volumes in Spain and the United States. The corresponding worldwide geographic heat maps of real-world COVID-19 confirmed cases (Figure 3) and deaths (Figure 4) provide visual comparative representations of these observations with the GT results. Figure 5A shows the sequence charts for the disease nomenclature searches. Of the GT disease nomenclature evaluated, the real-world (RW) confirmed cases and deaths were strongly correlated with COVID-19, coronavirus (virus; *r*=0.62, *r*=0.57, respectively), coronavirus (search term), and SARS-CoV-2 (search term; *r*=0.73, *r*=0.67, respectively). All these correlations demonstrated *P*<.001. Worldwide RW data were not statistically significantly correlated with severe acute respiratory syndrome (disease).

A total of 15 of the symptom search terms had statistically significant correlation coefficients with worldwide GT COVID-19, GT coronavirus (disease), and RW confirmed cases (Table 1). Of the included terms, only diarrhea failed to reach statistically significant correlation with RW deaths of COVID-19. The symptoms of shortness of breath (SOB), anosmia, ageusia, headache, chest pain, and sneezing all had strong correlations (*r*>0.60) to both new cases and deaths.

**Table 1:** Correlations among Google and Baidu search engines and real-world cases and deaths of COVID-19.

Search term^a^		WW^b^ cases	WW deaths	China cases	China deaths	Italy cases	Italy deaths	Spain cases	Spain deaths	WA cases	WA deaths	NY cases	NY deaths
**Real world deaths**													
	*r*	*0.87^c^*	N/A^d^	0.63	N/A	*0.95*	N/A	*0.97*	N/A	*0.92*	N/A	*0.87*	N/A
	*P* value	<.001	N/A	<.001	N/A	<.001	N/A	<.001	N/A	<.001	N/A	<.001	N/A
**Coronavirus**													
	*r*	*0.61*	0.56	0.35	0.049	0.33	0.19	0.44	0.32	*0.92*	*0.85*	*0.62*	0.51
	*P* value	<.001	<.001	.002	.67	.006	.12	<.001	<.001	<.001	<.001	<.001	<.001
**COVID-19 ^e^**													
	*r*	*0.82*	*0.75*	–0.20	–0.34	*0.95*	*0.87*	*0.86*	*0.77*	*0.89*	*0.84*	0.56	0.27
	*P* value	<.001	<.001	.08	.002	<.001	<.001	<.001	<.001	<.001	<.001	<.001	.11
**Fever**													
	*r*	0.41	0.33	0.47	–0.065	0.28	0.07	0.21	0.06	*0.83*	*0.84*	0.49	0.20
	*P* value	<.001	<.001	<.001	.57	.02	.56	.09	.62	<.001	<.001	<.001	.23
**SOB ^f^**													
	*r*	*0.73*	*0.65*	0.38	0.053	0.26	0.13	0.51	0.37	*0.76*	*0.73*	–0.11	–0.37
	*P* value	<.001	<.001	<.001	.65	.04	.31	<.001	<.001	<.001	<.001	.53	.03
**Cough**													
	*r*	0.35	0.26	0.56	0.33	–0.19	–0.37	0.21	0.05	0.46	0.54	–0.51	–0.65
	*P* value	<.001	.02	<.001	.003	.13	<.001	.08	.67	<.001	<.001	<.001	<.001
**Sputum**													
	*r*	0.48	0.39	0.48	0.32	0.05	–0.01	0.17	0.07	0.43	0.41	*0.63*	0.55
	*P* value	<.001	<.001	<.001	.005	.72	.92	.17	.58	<.001	<.001	<.001	<.001
**Anosmia**													
	*r*	*0.70*	*0.61*	–0.16	–0.21	*0.83*	*0.77*	0.58	0.47	*0.69*	0.58	*0.83*	0.53
	*P* value	<.001	<.001	.15	.06	<.001	<.001	<.001	<.001	<.001	<.001	<.001	<.001
**Dys/ageusia ^g^**													
	*r*	*0.75*	*0.66*	0.060	0.003	*0.68*	*0.64*	*0.69*	*0.58*	0.57	0.48	*0.94*	*0.73*
	*P* value	<.001	<.001	.60	.98	<.001	<.001	<.001	<.001	<.001	<.001	<.001	<.001
**Nasal congestion**													
	*r*	0.41	0.32	0.59	0.14	0.02	–0.04	0.08	–0.08	0.26	0.25	0.27	–0.05
	*P* value	<.001	.002	<.001	.24	.88	.74	.51	.53	.03	.03	.11	.77
**Rhinorrhea**													
	*r*	0.34	0.26	0.52	0.016	*0.64*	0.48	0.09	–0.02	*0.60*	0.57	0.50	0.40
	*P* value	<.001	.02	<.001	.16	<.001	<.001	.49	.86	<.001	<.001	<.001	.01
**Sneezing**													
	*r*	0.65	0.58	*0.78*	*0.73*	0.16	0.03	0.27	0.17	*0.65*	*0.69*	0.22	–0.04
	*P* value	<.001	<.001	<.001	<.001	.21	.81	.03	.18	<.001	<.001	.19	.82
**Sore throat**													
	*r*	0.49	0.41	*0.63*	0.34	–0.08	–0.17	0.27	0.11	0.29	0.38	–0.23	–0.32
	*P* value	<.001	<.001	<.001	.003	.55	.17	.03	.36	.01	<.001	.18	.05
**Headache**													
	*r*	*0.82*	*0.77*	0.56	*0.66*	0.16	0.09	0.46	0.35	0.18	0.20	–0.18	–0.43
	*P* value	<.001	<.001	<.001	<.001	.20	.46	<.001	<.001	.12	.08	.30	<.001
**Myalgia**													
	*r*	0.47	0.42	*0.64*	0.32	0.07	–0.07	0.42	0.31	0.24	0.17	–0.35	0.24
	*P* value	<.001	<.001	<.001	.005	.60	.56	<.001	.01	.04	.14	.03	.15
**Chest pain**													
	*r*	*0.83*	*0.75*	*0.80*	0.53	0.59	0.43	0.44	0.28	0.58	0.41	0.55	0.26
	*P* value	<.001	<.001	<.001	<.001	<.001	<.001	<.001	.02	<.001	<.001	<.001	.12
**Eye pain**													
	*r*	0.35	0.29	0.15	0.24	0.06	0.08	–0.05	–0.07	–0.12	–0.05	*0.81*	*0.61*
	*P* value	<.001	.006	.19	.03	.66	.50	.68	.57	.30	.64	<.001	<.001
**Diarrhea**													
	*r*	0.28	0.21	0.47	0.14	0.35	0.23	0.40	0.26	0.40	0.43	0.33	0.05
	*P* value	.008	.05	<.001	.21	.004	.06	<.001	.03	<.001	<.001	.05	.76

^a^Google Trends used for all regions excluding China. Baidu Index used for China.

^b^WW: worldwide.

^c^Italics denotes strong correlation of *r*>0.60.

^d^Not applicable.

^e^COVID-19: coronavirus disease.

^f^SOB: shortness of breath.

^g^Dysgeusia used for China and Baidu Index search. Ageusia used for all Google Trends searches.

### Chinese COVID-19 Data With Baidu Index

In China, the written phrase for coronavirus (冠状病毒) was the predominant term used for searches during the COVID-19 crisis. Searches for “coronavirus” were correlated with new Chinese cases of COVID-19 but were not correlated with deaths (Table 1). The term COVID-19 was introduced by the World Health Organization (WHO) on February 11, 2020, so searches for this term only started after the outbreak in China was well underway. Figure 5B plots the Baidu search volumes along with the RW Chinese confirmed cases and deaths. The symptoms that correlated with both new daily Chinese cases and deaths were cough (咳嗽), sputum (痰, 黏液), sneezing (喷嚏), sore throat (咽喉痛), myalgia (肌肉酸痛), chest pain (胸痛), and headache (头痛). Symptoms that correlated to new Chinese cases but not deaths were fever (发热, 发烧), shortness of breath (呼吸急促, 呼吸困难, 呼吸短难), nasal congestion (鼻塞), rhinorrhea (流鼻涕), and diarrhea (腹泻). Eye pain (眼痛) was the only symptom that correlated to deaths but not cases (Table 1). The symptoms with strong correlations (*r*>0.60) to new Chinese cases were sneezing, sore throat, myalgia, and chest pain. The symptoms with strong correlations to deaths in China were sneezing and headache.

### Italian and Spanish COVID-19 Data With Google Trends

Figure 5C is a sequence chart showing the geographic regional data. Spanish and Italian GT correlations are also displayed in Table 1. Symptoms strongly associated with new Italian cases (*r*>0.60) were anosmia, ageusia, rhinorrhea, and chest pain. Symptoms strongly correlated to Italian deaths (*r*>0.60) were anosmia and ageusia. Symptoms strongly associated with new Spanish cases (*r*>0.60) were anosmia and ageusia. The only symptom strongly correlated to Spanish deaths (*r*>0.60) was ageusia, though anosmia was the next closest (*r*=0.50).

### Washington and New York, United States COVID-19 Data With Google Trends

GT correlations with new daily cases are shown in Table 1. For Washington, fever, SOB, anosmia, rhinorrhea, and sneezing were strongly correlated with new in-state cases (*r*>0.60) though ageusia was close (*r*=0.58). Fever, SOB, rhinorrhea, and sneezing were strongly correlated with in-state deaths (*r*>0.60), though anosmia and ageusia were close with moderate correlations (*r*=0.58 and *r*=0.51, respectively). In New York, fever, sputum, anosmia, ageusia, rhinorrhea, chest pain, and eye pain correlated strongly with new in-state cases. Symptoms that correlated strongly to New York state deaths (*r*>0.60) were sputum, anosmia, and ageusia.

### Time Series Cross-Correlations With Lag

All time series were fit for the ARIMA models. Outliers were removed prior to the analysis from the worldwide and China's RW confirmed COVID-19 cases and deaths corresponding to February 13, 2020, in which a large amount of previously unreported cases was provided to the WHO on a single day [12].

**Figure 6:**
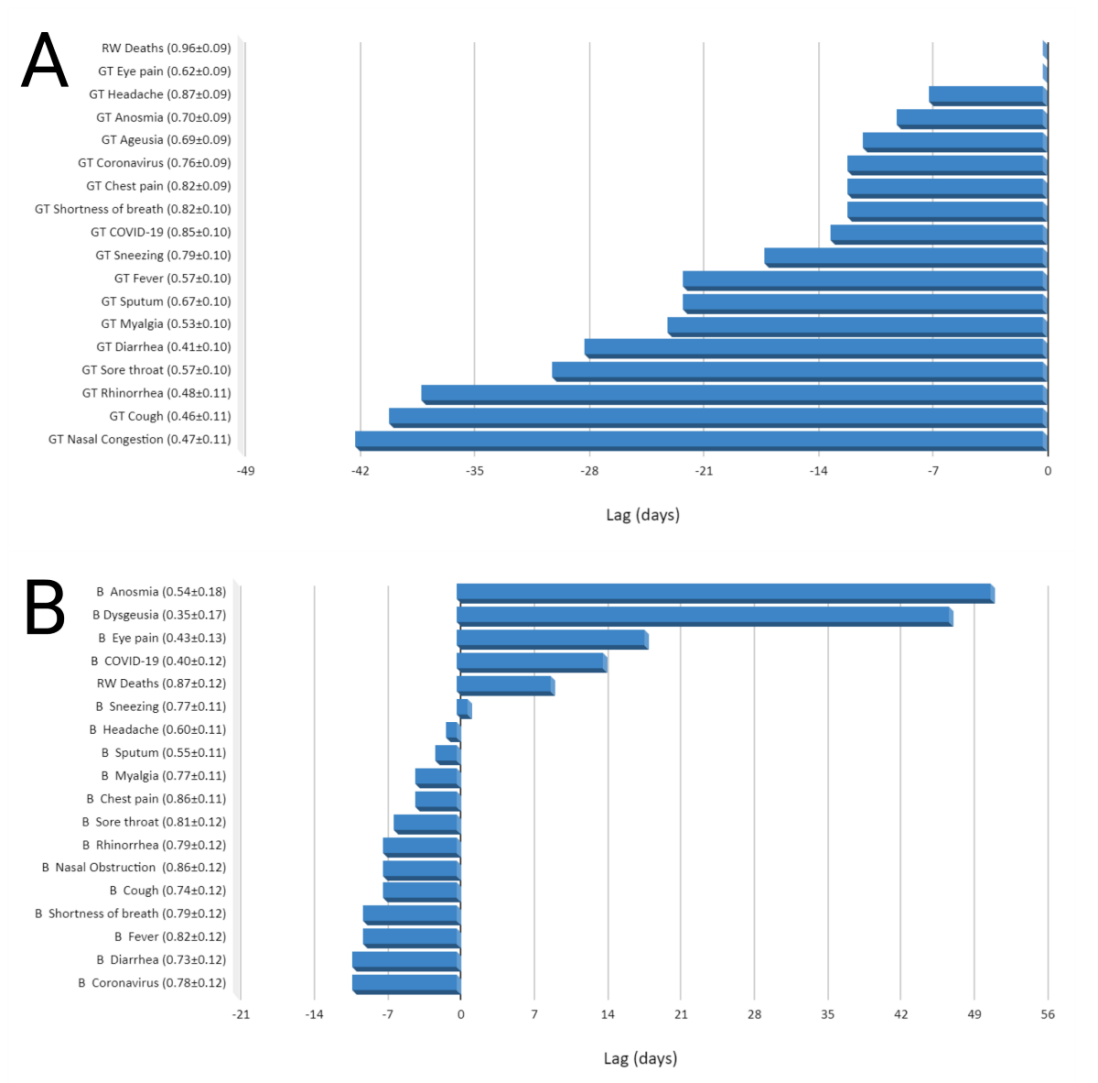
Lag correlations of online search terms worldwide to RW COVID-19 daily cases from January 9, 2020, to April 6, 2020. Note that a negative lag time means online searches preceded the daily RW cases. In parenthesis next to each search term is r±SE. (A) Lag-time of GT search terms, including GT coronavirus (virus), GT COVID-19 (search term), symptom term searches, and RW deaths compared to RW confirmed COVID-19 cases worldwide. (B) Lag time of Baidu Index search terms, including Baidu Index coronavirus (search term), Baidu Index COVID-19 (search term), symptom term searches, and RW Chinese deaths compared to RW confirmed COVID-19 cases in China. B: Baidu Index; COVID-19: coronavirus disease; GT: Google Trends; RW: real-world.

Figure 6 summarizes the lagged correlations in CCFs of the ARIMA models. As shown in Figure 6A, GT coronavirus (virus) and GT COVID-19 (search term) searches predated RW confirmed cases by approximately 12 days with strong correlations (*r*=0.79, SE 0.09 and *r*=0.84, SE 0.10, respectively) predating them by 19 days. Figure 6B shows a visual representation of lag correlations of RW confirmed cases compared to BI searches in China. Searches for BI coronavirus had a strong correlation with RW confirmed cases with a negative lag of 10 days (*r*=0.78, SE 0.12), while BI COVID-19 had a moderate correlation with a positive lag of 14 days (*r*=0.40, SE 0.12). Lag correlation of the various search terms and real-world cases demonstrated significant correlations with all terms (Figure 6B). Searches for symptoms of diarrhea, fever, shortness of breath, cough, nasal obstruction, and rhinorrhea all had negative lag >1 week compared to new daily cases.

GT anosmia and ageusia demonstrated very strong correlations with RW COVID-19 confirmed cases worldwide at a lag of 5 days, while Baidu searches for anosmia and dysgeusia had moderate to high correlations with RW COVID-19 confirmed cases in China at an extended lag of 64 and 57 days, respectively.

## Discussion

### Principal Findings

Our study demonstrates that digital epidemiology of the COVID-19 pandemic accurately correlated symptom searches around the globe with real-world cases and deaths, with internet searches preceding real-world cases and deaths by several days to a few weeks (Figure 6A). This lag time may represent a reporting bias, rooted in delays in testing [37]. Peaks of confirmed cases and deaths were similar, possibly due to the confirmation of COVID-19 status late in the disease course, closer to time of death. This lag time bias further justifies the importance of pursuing more real time assessments of disease development, ostensibly when people turn to the internet as they develop symptoms [24]. Previous epidemics have supported the use of internet searches for outbreak surveillance, suggesting that this method of surveillance may deserve more investment by public health agencies with development for the sole purposes of health care [4-7,9].

As SARS-CoV-2 is a novel virus, symptom constellation was poorly defined at the beginning of the outbreak. Symptoms evolved to include nasal congestion, sore throat, diarrhea, dysgeusia, and anosmia. Conceivably, digital epidemiology could assess these trended disease symptom searches in real time, actively correlating searches with real-world cases and deaths. Focusing on symptoms with strong correlations could then be emphasized in screening exams and public health campaigns.

With the evolution of anosmia as a recognized symptom, media influence was readily apparent. The first report on anosmia coinciding with the outbreak was published by Iran on March 9, 2020, though it did not disseminate internationally [30]. It was not until March 20, 2020 that the international medical community and mass media both began circulating press releases on the loss of smell as a potential marker for the COVID-19 infection [32,33]. GT searches for anosmia and ageusia were strongly correlated with RW COVID-19 confirmed cases worldwide at a lag of 5 days, while Baidu anosmia and dysgeusia searches had moderate to high correlations with RW confirmed cases in China at an extended lag of 64 and 57 days, respectively. These findings suggest that the search volumes of these terms were related to an index event, in this case after the scientific and journalistic media announced anosmia as a symptom on March 20, 2020. Within this atmosphere of constant and increasing media coverage, it is important to recognize the effect the media has on public interest. Cervellin et al [38] evaluated Google Trends in 2017 in an effort to determine its reliability as a tool for epidemiology. They found that, although reliable, it is certainly influenced by media coverage, which raises concerns for the true impact of these disease symptoms. This is matched by our data seen with anosmia peaking much later than other symptoms (Figure 6A), around the time of this mass media coverage (Figure 1).

Both search terms for loss of smell and taste had positive lag in our ARIMA models for both worldwide and Chinese data (Figure 6B), meaning that peaks in searches occurred after peaks in new cases. Our data show an enormous spike in these searches right after the time the international news media began to produce articles detailing these previously rare symptoms. It is important to consider that, although some of these searches may derive from patients with symptoms, they were accentuated by media attention.

As researchers learned more about COVID-19, other symptoms lesser known to the lay public were also being discussed among the medical community. Chest pain, myalgia, headache, and eye pain have all been reported. Although these symptoms have not received wide media coverage, they are consistent with recently discovered clinical manifestations of the disease, such as cardiac injury, embolic events, and neurologic sequelae [25,39,40]. In this study, these lesser known symptoms had similar lag times without the concern for media bias as seen with anosmia and other publicized symptoms. These symptoms may better represent patients developing disease, rather than those simply curious about the virus and its symptoms [41,42].

Though worldwide evaluation of cases and deaths provides data regarding the symptom profile of the disease, isolating regional data yields information about cultural differences, effects of the media, and of possible “super-spreader” events that could be used by public health officials as a form of contact tracing. The analysis of the Chinese BI data allows us to analyze the COVID-19 pandemic before the international medical community and media attention had the potential to distort search trends. The two symptoms that were correlated with new cases and deaths in China, sneezing and chest pain, were the two most frequently correlated symptoms to new cases and deaths in all regions studied. Dysgeusia was not found to be significantly correlated when analyzing China as a whole but was significantly correlated with new cases in Wuhan, the epicenter of the pandemic (*r*=0.22, *P*=.49). The significance of this finding, which manifested well before any known association between smell and taste loss with COVID-19, highlights the ability for informatics to identify the spread of disease using novel symptoms.

Lag correlation with ARIMA modeling did demonstrate significant correlation between new daily cases in China with anosmia and dysgeusia, but the lag was 64 and 57 days, respectively. This precisely corresponds to the increase in searches spurred from the announcement of these symptoms’ associations with COVID-19 in the media in late March [32,33,43,44]. This further highlights the potential for the media’s effects on this type of methodology.

Symptoms with negative lag have the potential for predicting location or size of disease outbreaks before they happen. In China, symptoms of rhinorrhea, nasal congestion, cough, shortness of breath, fever, and diarrhea all had significant lags of a week or more when correlated with new cases (Figure 6B). The media and medical community have paid significant attention to certain symptoms like fever, cough, and SOB, and these symptoms showed strong correlation (*r*>0.60) in the ARIMA modeling, confirming they could be good predictors for outbreaks. Interestingly, diarrhea is also a strongly correlated symptom with longer negative lag, indicating the potential for predictive values. Italy and Spain had their first confirmed cases of COVID-19 on January 31, 2020, and by mid-March, both countries were in full quarantine and had ceased all nonessential activity. The symptoms found to correlate to new cases and deaths in these two regions (Table 1) match well with our findings that the symptoms that most correlate with worldwide cases and deaths include headache, chest pain, sneezing, anosmia, and ageusia. Interestingly, our data also showed a direct correlation of search volume with major events within Italy and Spain. On February 19 in the Lombardy Region of Italy, 40,000 people attended a Champions league soccer match [27,29]. Similarly, on March 8, both an International Women’s Day March and a Vox party rally were taking place with thousands of people in attendance. As is apparent in Figure 5C, a peak in searches is seen directly after these dates.

In the United States, Washington was the first state to announce a COVID-19 case on January 21, 2020, and peaked with daily confirmed cases on March 23. New York then became the epicenter of the COVID-19 pandemic in the United States with one-third of the country’s cases. GT coronavirus (virus) and GT COVID-19 (search term) searches in these regions were strongly correlated with their respective regional RW confirmed cases and RW deaths. Analysis of GT for New York showed very strong correlations for both anosmia and ageusia with regard to daily confirmed cases and deaths, respectively. In this novel pandemic, this finding may demonstrate that, although media coverage may have the potential to distort the prevalence of certain disease characteristics, it may also be able to emphasize certain unique qualities of a disease once they have been identified. Interestingly, eye pain was a symptom that was found to be strongly correlated with new cases in New York, and this symptom was one that was found to correlate with new cases worldwide as well [25]. Eye pain has not had nearly as much media attention as loss of smell or taste, and it was added to a list of potential symptoms from less disseminated publications [25]. This may imply that further attention be paid to ophthalmologic complaints (eg, conjunctivitis) or headaches in this outbreak. With the use of Big Data such as Baidu and GT, there are limitations that must be acknowledged. Both platforms do not provide the exact methodology by which they generate search data, and the study population responsible for the searches cannot be determined [21]. The most widely discussed limitation is that search volumes can be heavily influenced by the dissemination of information through the internet or news media. Previous studies have also highlighted this limitation, and GT and BI may have better reliability defining the epidemiology for common diseases with minor media coverage or rare diseases and conditions with higher audiences. This was observed in our study with better reliability seen in those symptoms of COVID-19 with less media coverage [38,45].

### Conclusion

This study demonstrates the utility of digital epidemiology in providing helpful surveillance data of disease outbreaks like COVID-19. Although certain online search trends for this disease were influenced by media coverage, many search terms reflected clinical manifestations of the disease and showed strong correlations with RW cases and deaths.

## Appendix

Multimedia Appendix 1 Complete list of considered search terms.

## Abbreviations

ARIMA: autoregressive integrated moving average
BI: Baidu Index
CCF: cross-correlation functions
COVID-19: coronavirus disease
GT: Google Trends
RW: real-world
SARS: severe acute respiratory syndrome
SARS-CoV-2: severe acute respiratory syndrome coronavirus 2
SOB: shortness of breath
WHO: World Health Organization
