# Supplementary material for: Correlations of Online Search Engine Trends With Coronavirus Disease (COVID-19) Incidence: Infodemiology Study
Source: JMIR Public Health Surveill. 2020 May 21;6(2):e19702. doi: 10.2196/19702 (PMC7244220; doi:10.2196/19702)
Supplement: Multimedia Appendix 1 [file publichealth_v6i2e19702_app1.pdf]

Appendix 1. Complete list of considered search terms

**COVID-19**<br><br>Coronavirus (Virus, search term)<br>COVID-19 (search term)<br>SARS-CoV-2 (search term)<br>Severe acute respiratory syndrome (Disease)<br><br>**Upper Airway Symptoms**<br><br>Anosmia (Topic, search term)<br>Ageusia (Topic, search term)<br>Dizziness (Medical Condition, search term)<br>Dysgeusia (Disease, Topic, search term)<br>Nasal congestion (Syndrome, search term)<br>Rhinorrhea (Medical Condition, search term)<br>Sneeze (Topic, search term)<br>Sneezing (search term)<br>Sore throat (Topic, search term)<br><br>**Lower Airway Symptoms**<br><br>Cough (Disease, search term)<br>Dyspnea (search term)<br>Shortness of breath (Disease, Topic, search term)<br>Sputum (Topic, search term)	**Gastrointestinal Symptoms**<br><br>Abdominal pain (Syndrome, search term)<br>Diarrhea (Topic, search term)<br>Nausea (Disorder, search term)<br>Vomiting (Ailment, search term)<br><br>**Systemic Symptoms**<br><br>Anorexia (Symptom, search term)<br>Fatigue (Medical Condition, search term)<br>Fever<br><br>**Embolic Pain**<br><br>Chest pain (Syndrome, search term)<br>Headache (Medical Condition, search term)<br>Myalgia (Topic, search term)<br><br>**Other**<br><br>Eye pain (Topic, search term)<br>Skin rash (Disease, search term)
